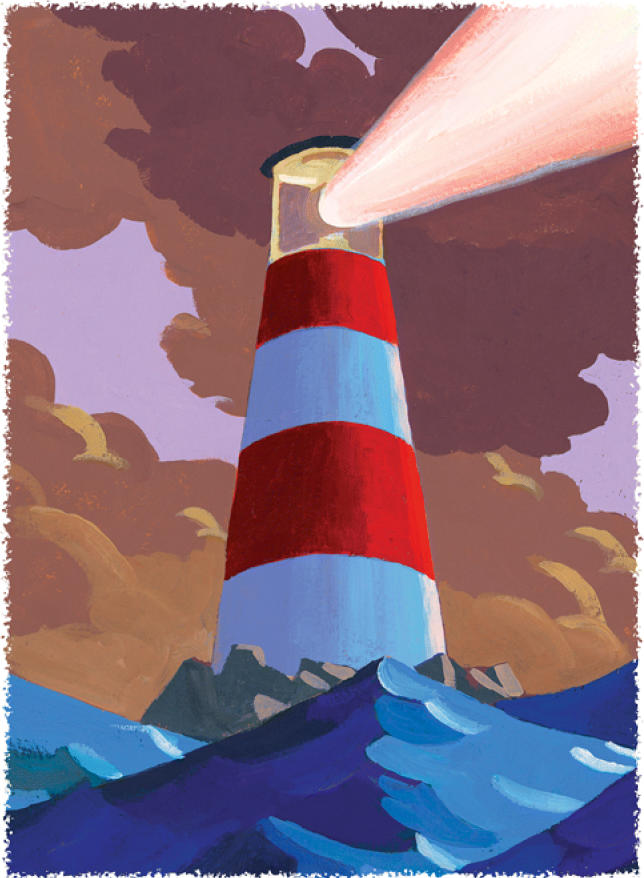# The Great Lakes Awash in Policies

**DOI:** 10.1289/ehp.113-a174

**Published:** 2005-03

**Authors:** Tina Adler

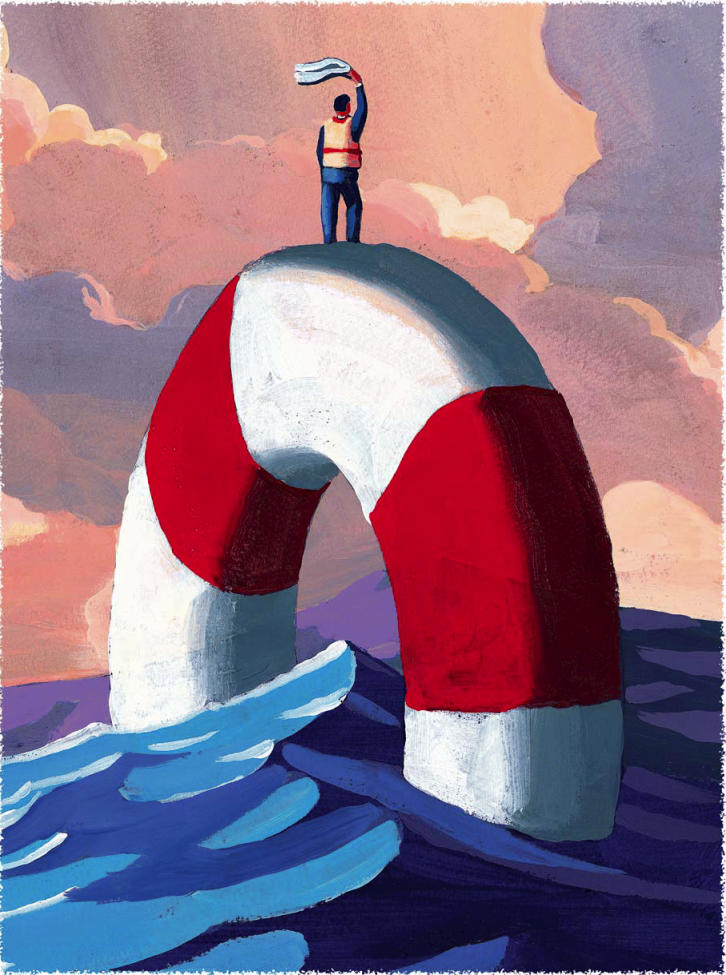



When a national resource has 8,300 miles of shoreline and 6 quadrillion gallons of fresh water—making it the largest surface freshwater system on Earth—it’s bound to attract some attention. Now surround that resource with eight states, two Canadian provinces, and multiple tribal lands, and you’ve got a political hot spot known as the Great Lakes basin. Add to this picture vast numbers of individuals and industries relying on the water to serve as their fishing—and dumping—grounds as well as a source of drinking water, transportation, recreation, and power, and it’s no wonder the U.S. government alone has about 140 programs devoted to the care and maintenance of the Great Lakes.

The Great Lakes basin has suffered from severe pollution problems, one of the most dramatic being recurring fires on one of Lake Erie’s arteries, the Cuyahoga River. The fires began in 1936, when a spark from a blowtorch ignited waste oil floating on the river. Recurrent fires continued until the early 1970s, when policy makers and others decided to crack down on pollution.

Nowadays, the fires are history and the lakes are cleaner. But the Great Lakes remain plagued by mercury contamination, legal and illegal dumping of industrial chemicals, burgeoning populations of invasive species, and dwindling food supplies and habitat for native creatures. The Environmental Protection Agency’s (EPA) *National Coastal Condition Report II*, released in January 2005, ranked the health of the lakes’ coastal waters between poor and fair, based on the deterioration of coastal wetlands, the poor condition of the lake bottoms, low levels of dissolved oxygen, and sediment contamination.

After 30 years of new policies, regulations, procedures, guidelines, agreements, and directives aimed at helping the lakes, old problems persist, and new ones are cropping up. Policy makers have determined that the Great Lakes are suffering from good, but very disorganized, intentions and a shortfall in funding. As former EPA administrator Mike Leavitt said in a 2004 speech, “We have lots of musicians, but we need more harmony.”

## Finding Harmony

In May 2004, in an effort to support restoration efforts and, some analysts say, create political goodwill in key election states during an election year, President Bush jumped into the Great Lakes policy arena. He issued an executive order that recognized the Great Lakes as a “national treasure” and established an interagency task force of 10 cabinet and agency heads to coordinate restoration projects under the EPA’s leadership.

The president also directed the EPA to convene a Great Lakes Regional Collaboration (GLRC) of stakeholders in the lakes. Participants held their first meeting on 3 December 2004. The collaboration includes elected officials from the eight Great Lakes states (Illinois, Indiana, Michigan, Minnesota, New York, Ohio, Pennsylvania, and Wisconsin) and representatives of municipal and county governments, environmental groups, and 30 Indian tribes. The Canadian provinces of Québec and Ontario, as well as the Government of Canada, serve as observers.

The collaboration, although intended to bring harmony to a discordant orchestra of voices and opinions, is “unprecedented in its scale and bureaucratic complexity,” the *Christian Science Monitor* asserted in its 22 December 2004 edition. Nevertheless, the collaboration “is coming together very well,” says Gary Gulezian, director of the EPA’s Great Lakes National Program Office in Chicago. The collaboration is “an idea whose time had come,” he asserts.

Most of the work of the collaboration is being done through eight “strategy teams.” Any representative of groups working on Great Lakes issues may volunteer to be on a team. Each team is addressing one priority issue: habitat and species, indicators and information, areas of concern, reduction of persistent bioaccumulative toxics, invasive species, sustainable development, coastal health, and non–point source pollution. These priority issues were endorsed by the Council of Great Lakes Governors (CGLG), a partnership formed to facilitate environmentally responsible economic growth. Each team is developing draft recommendations on how to make progress in its respective issue area. The recommendations will be publicly released in summer 2005 and finalized by the end of the year.

Part of the impetus for the collaboration was a 2003 report by the Government Accountability Office (GAO), then known as the General Accounting Office. The report asserted that restoration efforts for the Great Lakes lack leadership and organization, and require a comprehensive strategy similar to ecosystem restoration projects in South Florida and the Chesapeake Bay. “The GAO report really sparked things,” says Andy Buchsbaum, director of the National Wildlife Federation’s Great Lakes Natural Resource Center in Ann Arbor, Michigan, and a member of three of the strategy teams. A flurry of congressional hearings on the Great Lakes and the CGLG’s list of restoration priorities followed the publication of the report.

Buchsbaum and other Great Lakes experts describe the collaboration and cabinet-level task force as both unique and beneficial. Other policy initiatives either have focused on single issues, were limited geographically, or had few collaborators, he notes. Moreover, the EPA has put a lot of muscle into the collaboration and task force, Buchsbaum says.

That muscle was Leavitt, who took a personal interest in the lakes. He toured the Great Lakes states last year to discuss the environmental problems and brought those concerns cabinet-level attention. However, Leavitt’s involvement may be short-lived, as he was confirmed as secretary of the Department of Health and Human Services on 26 January 2005.

What his departure means to the collaboration and the task force remains unclear. “It is a cause for concern,” says Buchsbaum. “The process has been pretty self-sustaining to a large degree, at least for the first few months, but we don’t know if that will continue without Leavitt’s involvement.” He adds, “The big question is, at this time next year will we be able to say that this process has built momentum toward Great Lakes restoration? Or will it have simply distracted the players and stalled the momentum?”

The collaboration does have international support. Canada has representatives on all of the strategy teams established at the December collaboration meeting, says Marie-Christine Lilkoff, a spokesperson for the Canadian Department of Foreign Affairs. In a speech at the December meeting, Canadian consul general Anne Charles said, “The government of Canada welcomes the establishment of the U.S. Interagency Task Force and actions to improve coordination and strategic direction on Great Lakes policy, priorities, and programs in the United States, and to collaborate with Canada.” And in a press release issued when the task force was announced, David Anderson, Canada’s minister of the environment at the time, called the executive order “a strong sign of the importance of the Great Lakes to the United States government.”

## Cutting Out or Prioritizing?

However, the task force’s goal to prioritize restoration efforts is giving some members of the Great Lakes community the jitters. Industry representatives recognize the importance of gaining a better understanding of “where we are going and what we are trying to do,” says George Kuper, president of the Council of Great Lakes Industries in Ann Arbor, whose group includes Canadian and U.S. businesses. At the same time, Kuper, who is serving on two of the collaboration strategy teams, worries that the task force may inadvertently divert resources from successful efforts already under way, such as the Great Lakes Binational Toxics Strategy (GLBTS).

The GLBTS is a Canadian–U.S. agreement to work toward virtual elimination of 12 toxic, persistent, bioaccumulative substances from the Great Lakes basin and to reduce levels of an additional 15 substances from the environment around the Great Lakes. The agreement leaves it up to companies to decide how to achieve these goals, Kuper says. Environmentalists don’t like that it’s voluntary, “but it’s the most efficient way of meeting the targets and time tables,” he asserts. “And it’s working—we’re on track to meet the GLBTS goals by or before the 2006 deadline.”

Another concern is that efforts to coordinate and prioritize during tight budget times might be an excuse by policy makers to do less rather than more, says James Zorn, a policy analyst with the Great Lakes Indian Fish and Wildlife Commission, which represents the interests of 11 Ojibwe tribes. Zorn cochairs the collaboration’s persistent bioaccumulative toxics strategy team.

What’s lacking in restoration efforts is not priority setting but substantive policy or legislation to control the problems, Zorn says. Finally, he says, the devil is in the details: “It’s a big step if these divergent groups can agree on the priorities, but the question is will they agree on the specifics?” At the same time, Zorn appreciates that “Congress is more likely to listen when tribes speak as part of a collaboration on such issues as protecting habitat and resources.”

In response to concerns that the prioritizing will be more of a pruning, Gulezian says that the strategy teams are taking the approach of looking at policies, programs, and procedures already in place, and identifying which are the most effective at meeting the collaboration’s goals. The outcome won’t necessarily mean more funding for priority issues or pet programs, but instead, he believes, better use of existing resources.

## Diverting Diversions

Managing the use of Great Lakes water supplies is another hot topic in the basin, and one that is being addressed outside of collaboration efforts. The Great Lakes governors and the premiers of Ontario and Québec are in the process of implementing water use principles outlined in the Great Lakes Charter Annex of 2001. The annex is a good-faith agreement signed by the Great Lakes governors and premiers” the basin’s water supplies. Since signing the annex, the governors and premiers have been developing plans—or “implementing agreements”—to turn the principles outlined in the annex into legally binding standards.

The governors and premiers, in consultation with an advisory team of representatives from industrial, agricultural, municipal water supply, shipping, and environmental groups, are developing a management plan for regulating water diversions and withdrawals. The goal is to create uniform water management standards based on the annex principles for the states and provinces.

The central concern of the agreements is water diversions, or the permanent removal of water from the lakes—for example, to supply drinking water to several towns. Among other directives, the draft implementing agreements call for states to use collective decision making when deciding on proposals for significant new or increased water uses. The draft agreements also require that the basin be improved by any new or increased diversion or significant use of water.

The CGLG itself has received about 10,000 public comments on the drafts of the implementing agreements since releasing them in July 2004 for public review, says Lisa Wojnarowski, a CGLG program associate. Additional comments went to state and provincial governments. The state and provincial staffs who developed the agreements are now revising the implementing agreements based on those comments. Their goal is to have revised versions to the governors and premiers by this summer, says Wojnarowski.

Critics say that the agreements fail to make precise, enforceable recommendations. “We feel the proposed agreements do not provide a sufficient level of protection of waters in the Great Lakes basin,” says Lilkoff. “All jurisdictions [should focus more on] the need for greater conservation measures to make more efficient use of this finite resource.”

The Walter & Duncan Gordon Foundation, a public policy foundation in Toronto, invited four water conservation experts to review the draft agreements, and they agreed that the conservation measures outlined do not go far enough, as they wrote in a report to the foundation. At the same time, the experts wrote, the agreements need to be much simpler with more clearly stated principles.

Buchsbaum, however, considers the standards outlined in the agreements unprecedented in their level of protection. He is “cautiously optimistic” about the future of the agreements, “because there is consensus over the principle that we need to take a stronger action over diversions,” he says. He would always like to see the standards be tighter, he says, “but the general framework is good.”

The controversy now is over who will sit at the table when water use proposals are being assessed, Buchsbaum says. Unlike standards for water use in other areas of the country, the Great Lakes standards would be guided by what’s good for the lakes’ ecosystem, instead of by local economic pressures or whoever managed to claim the water first.

Tribes in the Great Lakes basin argue that they have been excluded from the drafting process of the agreements, says Ann McCammon-Soltis, a policy analyst with the Great Lakes Indian Fish and Wildlife Commission. The draft agreements say that states must consult with tribes about all proposed diversions or withdrawals, but “mere consultation is insufficient,” McCammon-Soltis wrote in comments presented in October to the CGLG. “The states do not have unfettered discretion to authorize withdrawals or diversions that would adversely affect or undermine treaty-guaranteed rights,” she asserted.

Representatives of the CGLG and tribal leaders met at the end of January to discuss the tribes’ and Canadian First Nations’ grievances. The governors’ representatives made clear that the agreement will not abridge treaty rights, and that they understand the tribes’ concern about being part of the process, says Zorn. However, how much the government plans to seek tribal participation remains unclear. At the same time, tribal governments could interfere with the process if not included, he notes.

## Future Funds

The long-term effect of these recent policy initiatives is hard to predict. The outcome will depend, in part, on how advocates manage to compete during tight budget times for federal dollars. Congressional representatives from Great Lakes states plan to reintroduce legislation to boost federal funding for the Great Lakes. The Senate’s Great Lakes Environmental Restoration Act proposes $6 billion for the lakes over the next 10 years, up from roughly $700 million. A similar House bill seeks $4 billion over five years. With billions of dollars going to huge restoration efforts like those for the Chesapeake Bay and the Everglades, advocates in the north hope to garner a similar financial commitment to ensure the Great Lakes continue to live up to their name.

## Figures and Tables

**Figure:**